# AI-Driven *De Novo* Binder Design: From Structure Prediction to Closed-Loop Optimization

**DOI:** 10.34133/csbj.0070

**Published:** 2026-04-27

**Authors:** Xinhao Li, Zeyu Fan, Jiaping Yang, Ruoyu Jiang, Fangyu Cao, Jinqian Li, Dan Xie, Xuanlin Meng, Mingjuan Sun, Lianghua Wang

**Affiliations:** Department of Biochemistry and Molecular Biology, College of Basic Medical Sciences, Naval Medical University, Shanghai 200433, China.

## Abstract

Protein binders, including antibodies and diverse nonantibody scaffolds such as monobodies and designed ankyrin repeat proteins, have become central to targeted therapy, molecular diagnostics, biosensing, and synthetic biology. However, conventional binder discovery remains largely dominated by screening-based paradigms (immunization or in vitro display followed by iterative optimization), which are constrained by library size, experimental cost, and multiobjective developability requirements. Recent advances in structure prediction, protein language models, and diffusion-based generative modeling have established a standardized technology stack for artificial intelligence-driven *de novo* binder design. This review summarizes this stack through a practical workflow. First, we outline essential data resources and the role of complex structure prediction. Next, we detail generative backbone design and structure-conditioned sequence optimization. We then evaluate computational metrics and uncertainty management. Finally, we discuss integrating high-throughput experimental feedback into closed-loop optimization. We conclude by discussing key challenges, including induced fit, negative design, and dataset bias, as well as future directions toward end-to-end complex generation.

## Graphical Abstract

**Figure:**
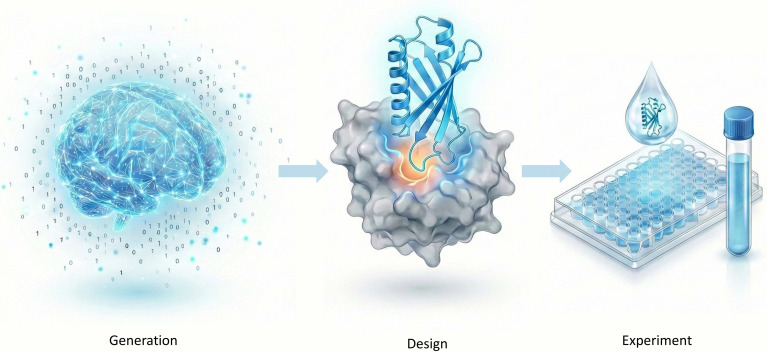

## Introduction

Protein–protein interactions form core network nodes in signal transduction, immune recognition, and metabolic regulation. The ability to intervene in these interfaces with exogenous and programmable molecules underpins modern biomedicine and synthetic biology [1]. Monoclonal antibodies have long dominated biologics because of their high affinity and specificity. However, their large size can limit tissue penetration and increase manufacturing and engineering costs. These limitations are especially pronounced in solid tumors, dense tissues, and diagnostic settings that require rapid clearance. Accordingly, a broad class of small binding proteins and nonantibody scaffolds (e.g., designed ankyrin repeat proteins, affibodies, and monobodies) has expanded rapidly over the past 2 decades [2,3]. These binders typically offer smaller size, greater engineering flexibility, and more straightforward expression in prokaryotic or cell-free systems, and they can be readily assembled into multispecific formats or embedded as imaging and sensing modules, enabling distinctive advantages in therapy, molecular imaging, in vitro diagnostics, and intracellular regulation [4–6].

**Fig. 1.**
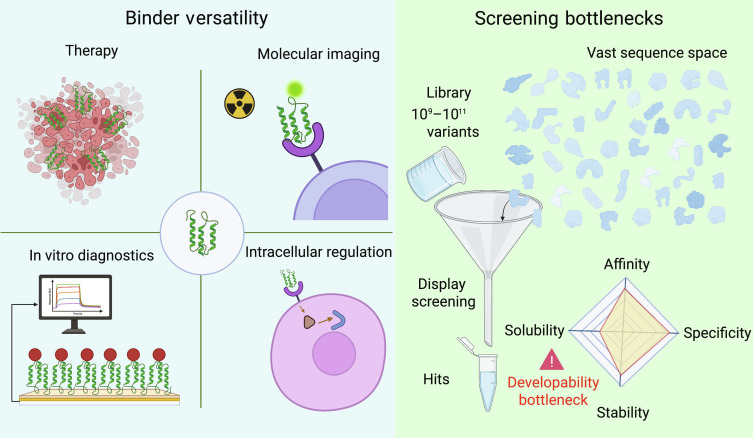
Binder versatility and the bottlenecks of traditional screening-based discovery. The left panel illustrates binder versatility, as protein binders enable diverse applications including therapy, molecular imaging, in vitro diagnostics, and intracellular regulation. The right panel highlights the developability constraints and bottlenecks of conventional discovery. This process typically relies on display-based library screening, where even large libraries (≈10^9^ to 10^11^ variants) sample only a tiny fraction of the astronomically large sequence space. As a result, screening often yields initial hits but faces a major developability bottleneck, as many candidates fail to simultaneously satisfy multiple biophysical and functional constraints. The radar plot conceptually depicts this challenge, representing the inherent multiobjective optimization and typical trade-offs across affinity, specificity, solubility, and stability required to obtain experimentally viable lead candidates (note: the radar plot is a conceptual schematic and is not derived from a specific dataset).

Despite strong application demand, mainstream binder discovery has long followed a paradigm of library construction, screening, and reoptimization. Animal immunization and in vitro display technologies (e.g., phage display and yeast surface display) identify binders by enriching massive variant libraries across multiple rounds of selection [7–9]. However, a fundamental bottleneck is the structural mismatch between the astronomical size of sequence space and finite experimental resources: even libraries on the order of 10^9^ to 10^11^ variants cover only a vanishingly small fraction of possible sequences. Consequently, screening can be particularly difficult for targets with flat or cryptic epitopes or for targets that require precise functional epitope constraints. Even libraries containing 10^9^ to 10^11^ variants may yield binders that show measurable binding but lack the desired function or exhibit poor developability. During lead optimization, affinity, specificity, stability, solubility, expression yield, and immunogenicity often need to be balanced against one another. This frequently leads to repeated cycles of directed evolution and increases both time and cost [10]. Figure 1 summarizes the versatility of protein binders alongside the key developability bottlenecks associated with traditional screening-based discovery.

**Fig. 2.**
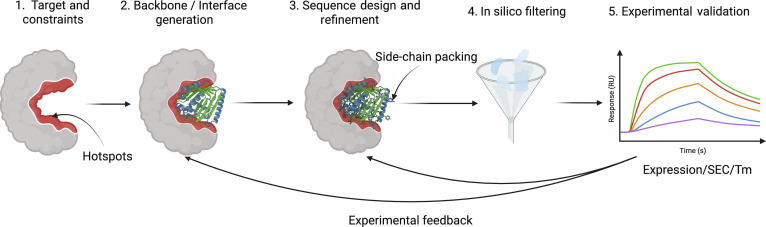
A standard AI-driven *de novo* binder design workflow. The pipeline starts from defining the target and design constraints (e.g., epitope and hotspots), followed by backbone and interface generation conditioned on the target. Candidate backbones are then converted into sequences and refined (e.g., inverse folding, side-chain packing, and refinement). Designs are prioritized through hierarchical in silico filtering, and a small set of candidates is advanced to experimental validation (e.g., expression, SEC, thermal stability, and biophysical binding assays). Experimental readouts are fed back to update the design and selection strategy, enabling iterative closed-loop optimization.

To overcome the scale and cost limitations of traditional library-and-screen paradigms, this review focuses on the rapidly maturing technology stack of artificial intelligence (AI)-driven *de novo* binder design. We begin with data resources and benchmarking practices, and then discuss the roles of monomer structure prediction and complex modeling in design workflows. Next, we review key methods for generative interface and backbone construction and structure-conditioned sequence design, followed by strategies for energy evaluation, uncertainty management, and engineering-grade in silico screening. We then describe how high-throughput experimental feedback can be incorporated into iterative closed-loop optimization. Finally, we summarize major bottlenecks, including induced fit, flexible interfaces, multiobjective developability, and standardized evaluation, and discuss future directions such as hybrid physics-informed generative systems and end-to-end complex generation. For orientation, Fig. 2 summarizes the standard AI-driven workflow for *de novo* binder design that will be used as the organizing backbone of this review, and Table 1 provides a compact toolbox index of representative modules and tools referenced throughout.

**Table 1.** Toolbox for AI-driven *de novo* binder design (compact view)

Stage	Core methods (examples)	Output and key points
1. Target and constraints	Target structure, epitope, hotspots Interface constraints (motif, orientation, contacts) Negative and off-target constraints	Design specification Constraint quality often dominates success
2. Backbone and interface generation	RFdiffusion All-Atom, Chroma, FrameDiff (motif scaffolding, inpainting) *De novo* generation (hallucination) Docking and pose proposal (unknown epitope) Co-folding, joint modeling	Backbone and pose ensemble Use multiseed sampling and clustering to preserve diversity
3. Sequence design and refinement	LigandMPNN, PiFold, SaProt (inverse folding and structure-aware LMs) Side-chain packing; constrained relax (Rosetta)	Sequence ensemble per backbone Remove clashes and preserve interface constraints
4. In silico filtering and ranking	Geometry gates (BSA, Sc, charge) Complex confidence (AF3, Chai-1, AF-Multimer ensemble) Energy and chemistry checks; Pose sanity (e.g., PoseBusters)	Cluster-based ranking with diverse representatives High confidence does not ensure high affinity; use consensus evaluation
5. Experimental + feedback	Expression, SEC, Tm Binding assays (SPR or BLI) HT display screens (optional) Active learning, model update	Validated hits and failure modes Use results to refine constraints and sampling

AI, artificial intelligence; LigandMPNN, ligand message passing neural network; LMs, language models; BSA, buried surface area; Sc, shape complementarity; AF3, AlphaFold 3; SEC, size-exclusion chromatography; SPR, surface plasmon resonance; BLI, biolayer interferometry; HT, high throughput

## Design Targets, Objectives, and Evaluation Metrics

### Types of *de novo* binders and common design settings

By the nature of the target, binder design can be broadly grouped into 3 settings: (a) protein targets, where binders modulate, block, or recruit protein–protein interactions; (b) small-molecule targets, where binders form complementary pockets or cavities to encapsulate the target; and (c) nucleic-acid targets, where binders engage DNA or RNA structures or motifs [11–13]. Different targets require different representations, constraints, and readouts. For protein surfaces, design usually focuses on epitopes and hotspot residues. For small-molecule targets, pocket geometry and ligand-atom constraints are more important. For nucleic-acid targets, binding-site geometry becomes a central consideration. These settings also differ in the readouts used to assess affinity, specificity, and developability [11,14–16]. Importantly, next-generation general-purpose modeling and generation methods are blurring these traditional boundaries: frameworks such as AlphaFold 3 (AF3) and related atomistic generative approaches increasingly support a unified all-atom view across protein–protein, protein–nucleic-acid, and protein–small-molecule interactions, while diffusion-based generation is being extended toward atom-level conditional design under complex constraints [17–19].

### Design objectives: Multidimensional trade-offs among affinity, specificity, and developability

At its core, binder design is a multiobjective optimization problem. Affinity is typically quantified by the equilibrium dissociation constant (*K*_D_) or by kinetic parameters (*k*_on_, *k*_off_), and structurally corresponds to geometric complementarity, buried surface area, and well-formed interaction networks (hydrogen bonds, salt bridges, and hydrophobic packing) at the interface [11,13]. Specificity further requires avoiding off-target binding and nonspecific adsorption, which may demand negative design and explicit consideration of competing states [20,21]. In practice, developability often determines whether a binder can enter a translational pipeline; key dimensions include thermal stability, solubility and expression yield, low aggregation propensity, and favorable physicochemical manufacturability. Industrial experience increasingly emphasizes that optimizing affinity alone is insufficient [22]. A practical strategy is therefore to front-load constraints into computation via a mix of hard constraints and soft penalties: for example, limiting unfavorable exposed hydrophobic patches and charge distributions during generation, and applying multicriteria gates during screening (structural confidence, geometric and energy-based metrics, and developability heuristics or predictors). Finally, high-information experimental readouts in closed-loop cycles can be used to calibrate weights and thresholds, reducing the risk of falling into single-objective optimization focused solely on affinity [10,23,24]. To bridge the gap between these abstract objectives and practical workflows, consider a concrete example: for affinity, computational surrogates like Rosetta interface energy or AlphaFold predicted aligned error are directly validated in vitro via yeast display or surface plasmon resonance (SPR). For specificity, in silico negative design scores or off-target docking penalties translate physically to rigorous counter-screening assays against homologous proteins. Finally, for developability, computational metrics such as the spatial aggregation propensity or net charge distributions are physically assessed using size-exclusion chromatography (SEC) for monodispersity and differential scanning fluorimetry (DSF) for thermal stability.

### Metrics and experimental readouts: A cascade from computation to experiments

Because model uncertainty is unavoidable, evaluation rarely reduces to a single definitive score; instead, robust pipelines rely on multiperspective agreement and tiered decision gates. To support hierarchical gating and multiview consistency checks, Table 2 summarizes representative computational metrics and experimental readouts, together with their typical use cases and caveats. On the computational side, the first layer focuses on interface geometry and plausibility: buried surface area, polar and hydrophobic complementarity, and hydrogen-bond and salt-bridge networks indicate whether an interface is physically reasonable; shape complementarity (Sc) is a classic metric that captures packing quality [25]. For overall complex quality, DockQ and related composite scores integrate multiple dimensions and facilitate cross-workflow comparisons [26]. The second layer uses stability and developability heuristics, together with energy-based checks, to flag aggregation and nonspecific risks. The third layer relies on predictor-derived confidence metrics for early filtering. Here, confidence (e.g., predicted local distance difference test [pLDDT] or interface predicted TM score [ipTM]) reflects the model’s internal reliability estimate, whereas accuracy refers to the agreement between predictions and experimentally determined structures. In protein complexes, pLDDT (local confidence), predicted aligned error (PAE) and interface predicted aligned error (iPAE) (uncertainty in inter-residue and inter-chain placement, especially across interfaces), and ipTM (interface-focused complex accuracy) are commonly used [27,28]. However, high computational confidence does not necessarily guarantee true in vitro binding, particularly for flexible interfaces, induced-fit systems, or out-of-distribution targets, where high-scoring false positives may still occur. Community assessments have highlighted both the proper scope and frequent misuse of AlphaFold 2 (AF2) confidence metrics [29] (see Table 3 for a detailed validation cascade). As AF3 and RoseTTAFold-All-Atom (RFAA) enter workflows, confidence metrics will extend to broader interaction types, but translating them into reproducible screening thresholds for binder design still requires systematic benchmarks and reproducibility studies [17,18]. Experimentally, workflows typically follow a fast-to-slow, coarse-to-fine cascade: rapid checks of expression, solubility, and thermostability; high-throughput binding and enrichment assays via phage or yeast display [7,9,10]; quantitative kinetics and *K*_D_ measurements by SPR or biolayer interferometry (BLI) to separate true high-affinity binders from nonspecific binders, aggregates, and surface-adsorbed species; SEC and aggregation analyses to assess monodispersity; and, for key projects, x-ray crystallography or cryo-electron microscopy (EM) structures to verify design intent at atomic resolution via root mean square deviation (RMSD) and interface-contact recapitulation. In practice, these objectives and evaluation metrics are connected through a staged design workflow. First, backbone generation, often driven by diffusion models such as RFdiffusion or Chroma, builds a geometrically complementary protein scaffold around the defined target epitope [12,30]. Second, sequence design or inverse folding (using models such as ProteinMPNN or Evolutionary Scale Modeling inverse folding (ESM-IF) optimizes an amino acid sequence to physically realize and stabilize that generated backbone [31]. Finally, structural validation employs the aforementioned confidence metrics (e.g., AF3 or ESMFold) to act as an in silico filter, ensuring that the designed sequence autonomously folds into the intended binding conformation before advancing to the experimental cascades outlined above [17,32]. This tight integration of generative algorithms with rigorous physical metrics is what enables the high success rates of contemporary *de novo* binder design.

**Table 2.** Key evaluation metrics and readouts (compact, nonredundant set)

Category	Metrics (examples)	Use	Pitfalls and notes
Interface geometry	BSA; shape complementarity (*S*_c_) Contact density, clashes	Fast early filters; remove bad poses	Good geometry does not ensure correct chemistry
Interface chemistry	Charge complementarity Hydrophobic patch, hotspot satisfaction	Reduce nonspecific and sticky interfaces	Heuristic; depends on residue modeling
Foldability, stability	Monomer pLDDT; core packing Predicted solubility and aggregation	Flag unstable or hard-to-express designs	pLDDT does not directly measure stability; predictors remain noisy
Complex confidence	ipTM; iPAE or PAE Interface pLDDT	Prioritize consistent complexes	High confidence does not ensure high affinity
Consensus and ensemble	Multirun AF; cluster RMSD Pose reproducibility	Filter single-run artifacts	Requires more computation; tune the number of runs
Physics-based ranking	Rosetta ΔG_int; H-bonds Clash and SASA terms	Rank within a cluster; refine top hits	Sensitive to relaxation settings; may overpack structures
Specificity and negative design	Off-target scoring, decoys Multistate evaluation	Lower cross-reactivity risk	Coverage is limited; select relevant decoys
Late-stage dynamics (optional)	Short MD; contact persistence Interface RMSD drift	Stress-test borderline candidates	Limited sampling depth; use selectively

BSA, buried surface area; pLDDT, predicted local distance difference test; ipTM, interface predicted TM score; iPAE, interface predicted aligned error; PAE, predicted aligned error; AF, AlphaFold; RMSD, root mean square deviation; SASA, solvent accessible surface area; MD, molecular dynamics

**Table 3.** Experimental validation hierarchy and a minimal validation set (aligned with the DBTL loop)

Validation level	Assays (examples)	Readouts	Why use	Notes
L0: expression and purity	Small-scale expression; Ni-NTA; SDS-PAGE	Yield; purity; soluble fraction	Feasibility gate	Drop low-expression, insoluble designs early
L1: high-throughput binding screen	Phage or yeast display; HT flow or ELISA	Enrichment; binding fraction	Fast hit triage	Can be noisy; enrichment does not directly reflect affinity
L2: developability screen	SEC-HPLC; DSF (Tm); aggregation, polyreactivity	Monomer fraction; Tm; nonspecific binding	Select tractable leads	Affinity gains may hurt solubility and stability
L3: quantitative kinetics	SPR or BLI (± ITC)	*K*_D_; *k*_on_, *k*_off_; specificity vs. controls	Rank candidates and reduce risk	Consider avidity and mass-transport artifacts
L4: mechanism, structure (optional)	Competition and epitope mapping; functional assay X-ray, cryo-EM, NMR (if needed)	Epitope; MoA; complex structure	Final proof	Not required for every campaign

DBTL, design–build–test–learn; Ni-NTA, nickel nitrilotriacetic acid; SDS-PAGE, sodium dodecyl sulfate–polyacrylamide gel electrophoresis; HT, high throughput; ELISA, enzyme-linked immunosorbent assay; SEC-HPLC, size exclusion chromatography–high-performance liquid chromatography; DSF, differential scanning fluorimetry; SPR, surface plasmon resonance; BLI, biolayer interferometry; ITC, isothermal titration calorimetry; EM, electron microscopy; NMR, nuclear magnetic resonance; MoA, mechanism of action

## Data Resources and Benchmarking

### Complex-structure data and dataset bias

Experimental complex structures in the Protein Data Bank (PDB) remain the gold standard for learning and validating interaction geometry. However, PDB sampling is intrinsically biased toward systems that are easy to express, purify, crystallize, or otherwise determine structurally, and it is enriched for certain classes such as antibody–antigen complexes and high-affinity, rigid interactions [33]. These biases can distort both model training and downstream evaluation. Accordingly, benchmark construction should apply stricter de-redundancy and interface-similarity control: beyond sequence-identity filtering, it is important to prevent near-duplicate interfaces from crossing train and test boundaries, which would otherwise inflate performance estimates [34].

A rapidly expanding complementary resource is large-scale predicted-structure databases. The continuing growth of AlphaFold DB provides unprecedented coverage for monomer structures [35] and has motivated prediction distillation and related strategies that treat predicted structures as pseudo-labels. Nevertheless, predicted structures should be used with care: they may propagate systematic errors, and for design-critical tasks (e.g., complex interfaces and pocket geometry), experimental structures remain preferable whenever available. For evaluation, it is also important to adopt metrics that are independent of the training objective and to calibrate with a small, experimentally validated subset whenever possible [36].

When curating these structural datasets, it is critical to rely on true biological assemblies rather than crystal-packing contacts to avoid misleading interaction priors. Furthermore, while the rapid growth of cryo-EM has drastically expanded the repertoire of available structures, substantial selection bias remains: transient interactions and membrane protein complexes are still largely underrepresented. Interestingly, the expanding diversity of the PDB also creates opportunities to retrain foundational models on specific subfamilies. For instance, retraining MPNN (message passing neural network)-style models on thermophilic proteins (e.g., HyperMPNN) can inherently bias the network toward higher stability, driving greater specificity and developability in AI-generated binders [37]. Additionally, leveraging preexisting target structures allows for highly structure-guided approaches to narrow the design search space, a strategy notably applied in the computationally challenging domain of G protein coupled receptor (GPCR) binder design [38].

### Sources and noise in experimental labels: Δ*G* and ΔΔ*G*, deep mutational scanning, and display readouts

If structural data determine what is geometrically feasible, experimental labels determine whether models can learn constraints that reflect energetics and function. High-quality affinity and kinetic measurements (e.g., SPR and isothermal titration calorimetry (ITC) provide quantitative labels for binding energetics, while curated mutation datasets (e.g., ΔΔ*G* benchmarks) enable learning how sequence changes perturb interactions [39]. However, measurements are often heterogeneous across laboratories, constructs, buffer conditions, and assay protocols. Such experimental-condition differences should be treated as a nonnegligible noise source during modeling. In benchmarking, uncertainty should be reported or controlled via stratified comparisons, to avoid misattributing system differences to model improvements.

High-throughput experiments have become a key data interface for closed-loop optimization. Deep mutational scanning (DMS) coupled with display-based enrichment and sequencing readouts can measure the relative fitness of thousands of mutants in a single experiment, enabling dense local maps of sequence–function landscapes [23]. However, enrichment is not equivalent to binding free energy or affinity. Affinity refers to the equilibrium binding strength between a binder and its target (e.g., quantified by *K*_D_), whereas enrichment reflects the relative increase of a sequence during selection rounds and is influenced by multiple factors including expression, folding stability, and display efficiency. Thus, a drop in enrichment may reflect reduced affinity, reduced expression, or reduced stability [10]. In closed-loop modeling, robust strategies include treating DMS as a ranking or preference signal, using multitask learning or explicit noise models to disentangle confounded factors, and anchoring key positions with a small number of low-throughput, high-accuracy measurements to reduce the risk of learning developability effects as if they were affinity effects.

### Benchmarking and reproducibility recommendations: Blind tests, leakage prevention, and false-positive control

In protein learning tasks, the most common source of illusory performance gains is data leakage. Because proteins exhibit extensive homology and structural reuse, random splits or splits based only on sequence identity can allow near-duplicate structures or interfaces to appear on both sides of the train and test boundary, producing overly optimistic results [36]. For binder design and complex prediction, leakage can occur not only through sequence similarity but also through shared folds, repeated interface motifs, and template overlap [40]. A practical reproducibility checklist therefore includes the following: explicitly defining the prediction target and potential leakage paths; performing de-redundancy at both sequence and structure and interface levels [20,41]; reevaluating similarity distributions after splitting; and reporting performance under progressively stricter splits. Where feasible, topology-disjoint or fold-disjoint splits should be used so that test targets are distant from the training distribution in both fold and interface type, thereby providing a more faithful assessment of out-of-distribution generalization [42]. To promote fair comparison and reproducibility across studies, we recommend an explicit benchmarking playbook for the field: (a) Split protocols: Datasets should enforce fold-level splits (e.g., clustering by Class, Architecture, Topology, Homology or Structural Classification of Proteins superfamilies prior to splitting) or epitope-disjoint splits for binding tasks, while capping sequence identity below approximately 30% across the training and testing boundary. (b) Minimal standardized task suite: New methods should be evaluated across a spectrum of target difficulties, including rigid antigenic proteins (e.g., viral glycoproteins), shallow or featureless protein–protein interfaces, and highly flexible or conformationally heterogeneous targets (e.g., GPCRs). (c) Reporting standards: Benchmarks should report dataset size, computational filtering thresholds, confidence metrics, experimental validation counts where available, and estimated false-positive rates derived from orthogonal physics-based scoring, together with indicators of generative diversity such as mode collapse.

## Structure Prediction and Complex Modeling: Roles in Binder Design

### Monomer structure prediction for stability and foldability assessment

For *de novo* generated backbones, the ability to fold spontaneously is a prerequisite for any downstream complex modeling and interface optimization. In practice, designers often use self-consistency tests: an inverse-folding model (e.g., ProteinMPNN) assigns sequences to a backbone; a structure predictor (e.g., AF2 or ESMFold) then predicts the structure from the sequence; and the predicted structure is aligned back to the design to evaluate backbone RMSD and confidence [31,32]. This step filters out designs that cannot reliably realize the intended fold. Nevertheless, foldability alone is not sufficient for developability: even well-folded designs can fail due to aggregation, low expression, or poor solution behavior. Therefore, stability and developability checks should be introduced early. These include in silico assessments, such as hydrophobic patch exposure and solubility prediction, as well as wet-lab assays such as DSF and SEC. This helps prevent structurally sound but nondevelopable molecules from advancing too far in the pipeline [10]. For instance, a computationally favorable candidate might still exhibit poor behavior in SEC, emphasizing the need for experimental validation.

### Complex prediction and binding-pose screening

Once monomer foldability is acceptable, the key question becomes whether a design forms a plausible bound pose and interface. AF3 brings protein–protein, protein–nucleic-acid, and protein–small-molecule interactions into a more unified atomistic modeling framework, while RFAA and related approaches provide complementary complex-prediction capabilities [17,18]. However, AF3 is currently constrained by strict intellectual property limitations, which restrict its free use in commercial or unconstrained high-throughput design pipelines. To address this, the community has rapidly developed fully open-source, free-to-use alternative models with comparable capabilities, such as Chai-1 and Boltz-1 [43,44]. Furthermore, newer iterations like Boltz-2 have advanced the field by implementing a dedicated affinity prediction pipeline natively into their architecture, enabling more direct scoring and ranking of binder candidates [45]. In binder workflows, complex predictors are commonly used for 2 purposes: (a) pose validation, in which the predicted complex is examined to determine whether the designed binder docks to the intended epitope and preserves the intended contacts; and (b) confidence-based filtering, in which candidates are removed when their predicted complexes are unstable, inconsistent across runs, or weakly supported by confidence metrics (e.g., inter-chain PAE or ipTM-like scores). Traditional docking methods like ClusPro function as conformational sampling engines to enumerate diverse initial orientations [46]; given their reliance on rigid-body assumptions and simplified energy terms, they are best used in cascade with end-to-end models: generating diverse conformations first, then reranking using deep learning confidence metrics. DockQ provides a unified quality measure comparing predicted complexes to reference structures [26], enabling quantitative assessment when experimental structures are available. DockQ v2 extends this approach to multimers, nucleic acids, and small molecules [47]. When reference structures are absent, designers must rely on multirun consistency, interface plausibility, and calibrated confidence thresholds to control false positives [48].

## Generative Design: Generating and Optimizing Interfaces and Backbones

**Fig. 3.**
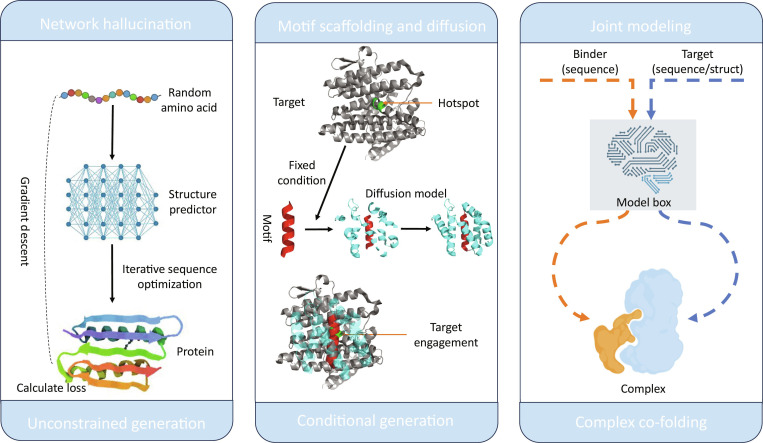
Comparison of 3 representative paradigms for protein design and complex prediction: network hallucination, conditional diffusion, and joint co-folding models. (Left) Network hallucination. The workflow begins with a random amino acid sequence. An iterative optimization cycle ensues using a structure predictor network. The network’s confidence or energy is used as a loss, which is then used in a gradient descent step to adjust the sequence. Through iterative sequence optimization, the network eventually predicts a novel, a well-folded protein structure. (Center) Motif scaffolding and diffusion. The design process is initiated with a known target protein (gray) featuring specific functional hotspots (green). This is set as a fixed condition. A functional motif (red) is then used with a diffusion model to scaffold a new protein structure around it. This method aims to generate a complete protein complex where the functional motif (red) is precisely engaged at the specified hotspot location of the target protein. (Right) Joint modeling. This approach uses a joint deep learning network model to predict the complex structure of 2 proteins simultaneously. The sequence of the binder and the sequence or structure of the target are fed into the model. The model directly predicts a single, complete complex structure resulting from the simultaneous co-folding of both molecules.

With the advent of controllable structure-level generative models, *de novo* binder design has shifted from retrieving shape-matched candidates from natural scaffold libraries to customizing novel scaffolds around the target’s geometric and chemical environment. The key enabler is that, whether through explicit SE(3)-equivariant architectures or extensive spatial data augmentation, these models effectively capture the fundamental geometric and physicochemical constraints governing 3-dimensional protein conformations and interface complementarity. Diffusion models, in particular, sample from a probability distribution over backbones that satisfy user-specified constraints, thereby dramatically expanding the accessible topology and interface-shape space [12,19]. This has become a mainstream entry point for generating binder backbones and interfaces. In parallel, a family of approaches that treat structure predictors as optimization objectives has also proven effective for binder design: from early network hallucination and inverse optimization ideas to more recent automated one-shot pipelines, these methods directly drive generation using interface confidence or energy-like objectives, shortening the engineering chain from target to candidate binders and increasing the probability of securing functional hits with reduced experimental screening [49,50]. To clarify commonly conflated generative paradigms, Fig. 3 summarizes 3 representative strategies: network hallucination, motif-conditioned diffusion design, and joint co-folding models.

### Target-conditioned backbone generation: Geometric constraints and objective matching

The core difficulty of generating binder backbones conditioned on a target surface is to satisfy 2 sets of constraints simultaneously: (a) the monomer must be foldable and expressible (backbone self-consistency), and (b) the interface must be complementary, forming a stable contact network at the desired epitope or region. Diffusion-based structure generation (e.g., RFdiffusion) models backbone generation as an iterative denoising and sampling process, and allows epitopes, hotspot residues, relative orientations, and contact-density constraints to be injected as conditions. This enables direct generation of interface backbones that are geometrically complementary to a target, and supports inpainting to complete local regions while preserving key interactions [12,19]. Beyond geometric conditioning, surface-learning methods that encode molecular-surface fingerprints can provide more physically relevant conditioning signals: curvature, hydrophobicity, and charge patterns can guide generation so that candidates are not only geometric proximity but also chemical complementarity, reducing geometrically plausible yet chemically implausible proposals [20,21]. Finally, engineering pipelines such as BindCraft further automate the end-to-end process by coupling generation and screening with predictor-based differentiable signals, illustrating a trend from demonstrating stand-alone model capabilities to establishing reusable and scalable design pipelines [50].

### Balancing hard and soft constraints in structure-conditioned design

Generative binder design typically involves both hard constraints that must be satisfied and soft constraints that are desirable. A canonical hard constraint is fixing motifs or hotspots: specific interface residues, secondary-structure elements, or functional motifs are required to maintain their relative conformations during sampling, while the model completes the remaining backbone [51]. Diffusion frameworks have developed systematic strategies for such motif scaffolding, improving functional-motif fidelity. Soft constraints are closer to practical engineering needs: interface area, Sc, hydrophobic and electrostatic matching, secondary-structure preferences, and penalties on flexible loops can be incorporated as guiding potentials so that sampling is steered toward more developable regions without being overconstrained. Surface-based models (e.g., dMaSIF-like fingerprints) can provide differentiable signals for interface chemical–geometric matching and are often used to operationalize such soft guidance [52]. In addition, sequence-level generative models can act as a constraint-integrating framework: discrete diffusion and related methods can introduce controllable edits and multiobjective preferences in sequence space (e.g., limiting exposed hydrophobicity, controlling charge distributions, or avoiding unwanted motifs), complementing structure diffusion and pushing developability constraints earlier in the pipeline rather than relying solely on post hoc filtering [53].

### Diversity and controllability: Multisolution sampling and avoiding mode collapse

A major advantage of generative design is the ability to sample multiple feasible solutions for the same target, yet in practice models can suffer from mode collapse (repeatedly producing a small number of common topologies such as helical bundles) [12]. Engineering practice typically maintains diversity via 3 approaches: (a) adjusting sampling temperature or noise schedules and performing multiseed restarts to balance high-confidence exploitation against exploration; (b) providing more fine-grained conditional signals (epitope, hotspots, or surface fingerprints) so that diversity reflects genuinely different ways of satisfying the same functional constraints rather than minor perturbations of one topology [12,19,21]; and (c) performing structure-based clustering and de-redundancy downstream, ensuring that candidates entering sequence design and filtering span multiple structural clusters [50]. Overall, generative models do not provide a single optimal solution, but rather a portfolio of structural hypotheses that can be exploited by downstream screening and closed-loop optimization.

### Exploring complex conformations: From docking proposals to conformational sampling

When the epitope is unknown or whole-surface exploration is required, unconditional or full-surface conditional generation can be computationally prohibitive. A common strategy is therefore initial pose generation followed by local structural refinement. In protein–ligand or pocket-related settings, models such as DiffDock and EquiBind can rapidly propose candidate binding sites and poses, serving as starting points for subsequent pocket remodeling or binder generation and compressing the search space from the entire surface to a handful of high-probability regions [54]. More advanced multiscale frameworks such as NeuralPLexer attempt to jointly model conformational changes of both protein and ligand and therefore partially incorporate induced fit into generation. This capability is particularly important when pocket rearrangements or flexible adaptation are required [55,56]. In protein–protein binder design, an analogous cascade is to localize potential epitopes using coarse docking or surface fingerprints, then grow interfaces locally via diffusion-based backbone generation, and finally filter candidates using complex prediction and uncertainty metrics, implementing a staged workflow comprising pose exploration, interface generation, and structural screening [19–21].

## Sequence Design and Refinement: From Backbone to Experimentally Tractable Molecules

After generative models produce a target-engaging geometric backbone, the practical success rate is often determined by whether that backbone can be turned into a real molecule that is expressible, purifiable, stable in solution, and retains the intended interface chemistry. The central task of sequence design is to assign amino acids to a fixed backbone (and any epitope or ligand-geometry constraints) so that the sequence simultaneously supports self-consistent folding, realizable interface interaction networks, and acceptable developability (solubility, aggregation propensity, thermostability, and expression yield). A key difficulty is that local optima can conflict. For example, increasing hydrophobic contact to improve affinity can also increase the risk of aggregation, whereas increasing core hydrophobicity to stabilize the fold may reduce expression or promote unwanted oligomerization. As a result, mainstream pipelines adopt a funnel strategy: inverse-folding models rapidly propose diverse sequence candidates; physics-based and all-atom refinement removes stereochemical clashes and improves packing; multistate evaluation and negative design eliminate cross-reactive or sticky candidates; and a small set is advanced to experimental closed-loop validation.

### Inverse folding: The workhorse for structure-conditioned sequence assignment

Inverse folding formalizes backbone-to-sequence as conditional probability modeling: given fixed backbone coordinates, the model predicts an amino acid distribution at each position and samples sequences accordingly [31]. In practice, inverse folding is used not only to produce a single best sequence but to generate a diverse panel for downstream screening. It also provides a natural handle for steering multiobjective preferences: for example, temperatures and biases can be tuned so that buried cores favor hydrophobic residues, while exposed surfaces are biased toward charged and polar residues to improve solubility and charge balance. This shifts part of the affinity–developability trade-off from late-stage empirical correction to the earlier sequence-assignment stage.

Beyond sequence generation, inverse-folding models can serve as learnable scorers. Sitewise log-likelihoods or perplexity provide calibrated signals for sequence–backbone compatibility, enabling rapid triage and identifying unstable or undersupported positions. Moreover, conditioning can be extended from monomer backbones to interface-aware contexts, allowing models to favor amino acids that realize specific contact patterns or hydrogen-bond geometries [57]. From a geometric-representation perspective, modules such as Geometric Vector Perceptrons process both scalar and vector features [58], improving sensitivity to orientation, torsion, and dense packing, all of which are critical for both foldability and interface quality [59].

Inverse-folding models are also being specialized for distinct molecular contexts. For ligand and pocket-related design, LigandMPNN explicitly models nonprotein atoms (ligands, metal ions, or cofactors) and improves sequence assignment around heterogeneous binding sites [60]. Analogously, antibody- or framework-specific inverse-folding models incorporate domain priors and constraints. Together, these developments push inverse folding from a general-purpose tool into specialized components that directly serve different binder classes (protein binders, ligand-binding proteins, and antibodies) [61].

### Emerging paradigms: Joint codesign and multimodal language models

The conventional workflow separates backbone generation from sequence design [31]. Although this strategy remains widely used, it can produce structures that are difficult to realize with sequences that fold robustly in practice. This limitation has increased interest in methods that couple structural and sequence information more directly during design. One line of work focuses on joint structure-sequence generation. For example, Chroma enables protein generation under programmable geometric constraints, while newer all-atom generative models extend this idea by incorporating finer structural detail during design [30,62]. These methods aim to reduce the mismatch between a generated backbone and the sequence needed to stabilize it. A second line of work comes from protein language models. Early generative models such as ProGen and ProtGPT2 showed that large-scale sequence training can support the generation of plausible protein sequences across diverse families [63,64]. More recent multimodal models, including the ESM series, further extend this framework by allowing conditioning on multiple information types, such as sequence, structure, and functional annotations [65]. In binder design, these models may be useful because they allow sequence generation to be informed by structural constraints and functional requirements at an earlier stage. Taken together, these studies suggest a gradual shift away from strictly sequential design pipelines toward more integrated workflows in which structural feasibility and sequence compatibility are considered together.

### Physical refinement: All-atom side-chain packing and energy consistency

Deep-learning sequence models are typically backbone-centered and cannot guarantee atom-level stereochemical correctness, optimal hydrogen-bond geometry, or tight packing of hydrophobic cores and interfaces. Therefore, before synthesis and testing, physics-based refinement is frequently employed as a standard validation step. All-atom energy functions (e.g., Rosetta REF2015) enable side-chain repacking and relaxation to remove clashes, improve packing, and repair strained geometries, providing an interpretable hard constraint layer [66,67]. Refinement can also be used to enforce developability preferences: for instance, improving solubility should generally prioritize redistributing charge and polarity on solvent-exposed surfaces away from the interface, rather than expanding interfacial hydrophobicity, thereby reducing the common failure mode of wherein enhanced affinity comes at the cost of exacerbated aggregation.

### Specificity and multistate design: Negative design, cross-docking, and multiconformation compatibility

For in vivo or clinical applications, the requirement is often not merely target engagement, but strict target specificity combined with stability in physiological environments. These requirements can be considered from 3 practical perspectives: negative design, multistate evaluation, and the balance between affinity and developability. (a) Negative and off-target selection: Systematically cross-dock candidate binders against structurally similar decoy targets and common nonspecific cellular surfaces. Apply explicit computational filters to penalize off-target binding propensity, nonspecific hydrophobic patches, and undesired oligomeric states [68]. (b) Multistate design for flexibility: For flexible targets and induced-fit systems, a single static structure is insufficient [69]. Evaluate candidates across a target’s conformational ensemble (e.g., bound versus unbound states) to ensure the binder selectively stabilizes the intended functional state and maintains multiconformation compatibility. (c) Reconciling conflicting objectives: To balance affinity against developability without collapsing metrics into a single scalar score too early, pipelines should maintain a Pareto frontier. Track interface energy independently from developability metrics (e.g., net charge and surface hydrophobicity) to preserve highly developable but moderately affine candidates for later experimental optimization. Finally, a small experimental calibration loop on a handful of candidates (e.g., alanine scanning, DMS at key sites, or competition assays) can quantify model biases and feed back into improved scoring and selection.

## Binding-Energy Evaluation and Prioritization: Uncertainty Management and Optimization Strategies

After the standard pipeline comprising backbone generation, sequence assignment, and structural validation, it is common to obtain thousands to tens of thousands of candidate binders, while experimental capacity typically allows validation of only tens to hundreds [70]. The goal of computational evaluation is therefore threefold: eliminate candidates that are unlikely to fold, bind, or be developable; provide a stable relative ranking for the remainder; and quantify uncertainty to control the false-positive rate so that a top-ranked computational score is more likely to correspond to a trustworthy decision.

### Data-driven interface scoring: Positioning and limits of Δ*G* and ΔΔ*G* prediction

Structure-based deep-learning scoring functions are often sensitive to interface geometric and chemical complementarity. Models such as ScanNet and DeepRank-GNN, a graph neural network framework, can identify interaction hotspots and provide useful ranking signals in large candidate sets [71,72]. However, their absolute calibration is limited by training data biases, label noise, and distribution shift [39], and they may not faithfully approximate physical binding free energies [73]. A safer and more robust practice is to treat data-driven scores as relative ranking cues or hotspot annotations, and to combine them with physics consistency checks and structural confidence rather than making decisions based on a single learned score.

### Physics-based energy functions and high-accuracy free-energy methods: Interpretable hard constraints

Physics-based energy functions remain the most reliable hard constraint in screening funnels, particularly for excluding steric clashes, exposed hydrophobics, and implausible buried charges. All-atom energy functions and refinement (e.g., Rosetta) are routinely used to repair stereochemistry and packing before experimental testing [66,67]. More accurate free-energy methods and molecular dynamics (MD)-based evaluations can further support mechanism-focused validation and reranking. However, these approaches are computationally expensive and sensitive to force fields and sampling adequacy, and are thus best reserved for late-stage verification and small-set prioritization rather than large-scale first-pass filtering [74].

### A practical screening funnel: From geometric gates to joint ranking

**Fig. 4.**
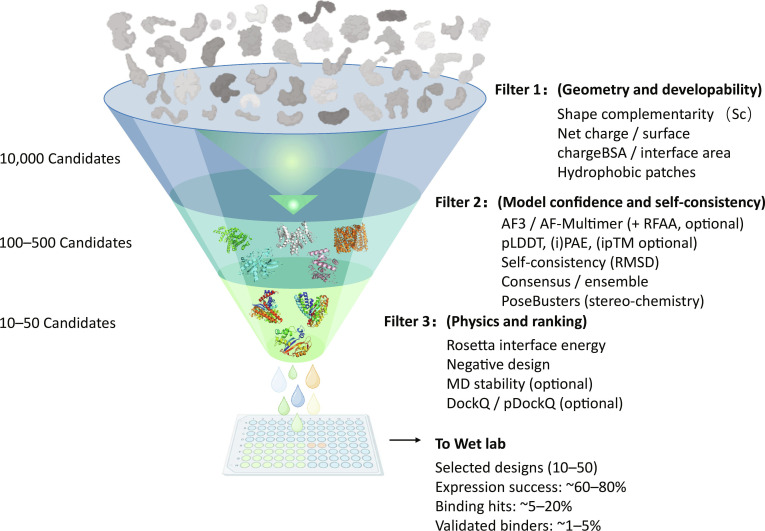
Hierarchical computational screening funnel for binder design. Large candidate sets are progressively reduced through a 3-layer filtering strategy. The top layer applies fast geometric heuristics and developability-related checks (e.g., shape complementarity [Sc], surface charge, and interface buried surface area). The middle layer removes false positives using deep-learning-based complex prediction confidence and structural self-consistency (e.g., pLDDT, (i)PAE, and pose stability across runs). The bottom layer refines and ranks candidates by physics-based metrics (e.g., Rosetta interface energy) and specificity-oriented negative design, optionally followed by late-stage stability checks (e.g., MD). The final set is handed off for experimental validation.

In practice, the most effective approach is to construct evaluation as a hierarchical funnel. Because design stages produce large candidate pools, practical pipelines use a hierarchical in silico screening funnel to reduce candidates to an experimentally tractable set (Fig. 4). The first layer uses geometric and developability heuristics. For instance, designers typically enforce Sc scores above 0.65, interface buried surface area between 1,200 and 2,000 Å^2^, and strict limits on solvent-exposed hydrophobic patches. This step aims to reduce downstream experimental failure rates by removing obviously problematic candidates early [22,25].

The second layer introduces end-to-end structure predictors for self-consistency checks and consensus and ensemble filtering. The goal is to verify whether sequences proposed by inverse folding reliably realize the designed fold and whether the predicted complex converges to a stable pose across multiple runs [19,31]. Key indicators include confidence metrics (e.g., demanding monomer pLDDT > 85, interface PAE < 5 Å, and ipTM > 0.8), pose stability, and RMSD consistency [17,18,28]. To avoid physically unrealistic predictions with inflated deep-learning scores, orthogonal stereochemical sanity checks can also be added. For example, PoseBusters-like checks of bond lengths, bond angles, and atomic clashes can help ensure basic conformational realism [75]. Even with these stringent in silico filters, designers should expect substantial false-positive rates: typical experimental hit rates for *de novo* binders currently range from 1% to 10%, occasionally reaching up to 20% for highly favorable targets, underscoring that computational scores enrich for success rather than guarantee it.

The third layer performs higher-precision joint ranking and pose quantification. In protein-only systems, physics-based Rosetta energy terms can be combined with learned interface scorers to improve robustness through 3-dimensional convolutional neural network or graph-based models [76,77]. For specificity, negative design and cross-docking against off-targets can be incorporated at this stage. Nevertheless, even sophisticated filters can yield false positives for induced-fit or highly flexible targets; MD simulations that explicitly model backbone motions and mutual conformational changes provide one strategy for better capturing dynamic binding interfaces, as demonstrated by dynamic docking approaches and multistructural docking combined with MD calculations [78,79].

### Uncertainty management in candidate prioritization

The key to screening decisions is not to chase a single highest score, but to control false positives arising from uncertainty. First, confidence metrics from structure predictors should be treated as reliability cues rather than physical ground truth, and their interpretation should be calibrated for each task [29,80]. Second, consensus across multiple seeds, ensembling across models, and stability under perturbations can provide practical robustness checks. Third, thresholds should be calibrated with small, high-quality experimental controls (e.g., a few SPR or BLI measurements, competition assays, or key-site mutations), and the resulting feedback should be incorporated into subsequent rounds of generation and sequence optimization to form a sustainable loop that progressively reduces false positives [81].

## Closed-Loop Optimization: Integrating Wet-Lab Feedback via DBTL and Active Learning

**Fig. 5.**
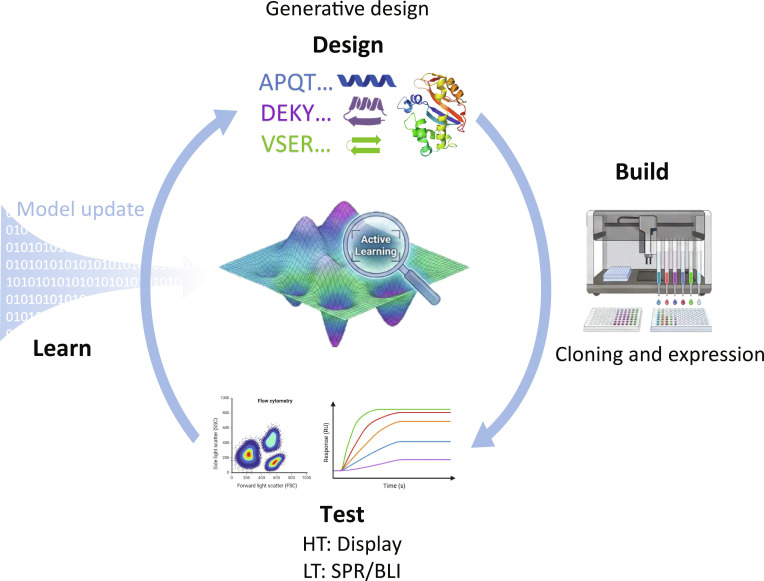
Closed-loop optimization for *de novo* binder design via design–build–test–learn (DBTL) and active learning. Candidate binders are proposed in the Design stage using generative models and sequence design, then constructed through Build (cloning and expression). In the Test stage, high-throughput assays (HT; display-based screening) provide large but noisy measurements, while low-throughput biophysical assays (LT; SPR or BLI) yield accurate affinity and kinetics for a smaller set of candidates. Experimental readouts are used in the Learn stage to update a surrogate model (model update) and guide the next design cycle via active learning, balancing exploration and exploitation to improve hit quality over iterations.

Although generative backbone and interface design and inverse-folding models can now produce large numbers of candidates at the structural level, systematic gaps remain between computational scores and real biophysical performance. Solvent effects, conformational dynamics, expression and folding constraints, and assay-dependent artifacts can all break the link between in silico objectives and experimental outcomes. Consequently, robust binder programs increasingly adopt design–build–test–learn (DBTL) cycles, where wet-lab readouts continuously recalibrate models and selection strategies [82–84]. Figure 5 summarizes a DBTL closed loop with active learning, where high-throughput assays provide large but noisy signals and low-throughput assays provide high-accuracy kinetic and developability measurements for a smaller set, enabling iterative improvement.

### Learning from high-throughput readouts: DMS, display enrichment, and noise-aware modeling

DMS enrichment signals are often confounded by expression and folding effects and are not directly equal to affinity. In closed-loop modeling, more robust strategies treat DMS as a ranking or preference signal rather than an absolute energy label, combine it with multitask learning or explicit noise models to separate latent factors (affinity, expression or stability), and anchor key sites with a small number of low-throughput, high-accuracy measurements [83–85]. Such models can then provide calibrated uncertainty estimates that feed into active learning or Bayesian optimization proposals, turning the constraints of data scarcity and experimental cost into a strategy of maximizing information gain per experimental round [82–84].

### Reinforcement learning and evolutionary algorithms for local optimization

Once an initial hit is obtained, even if its activity is weak, subsequent steps such as affinity maturation, specificity enhancement, or developability repair often benefit more from local search than from restarting generation from scratch. A concrete example in recent literature is the use of partial diffusion for structural refinement. In frameworks like RFdiffusion, researchers can “melt” and resample specific suboptimal binding loops by adding localized noise, and then progressively denoise them into higher-affinity conformations while strictly preserving the stable core scaffold of the lead binder [12]. In small-data regimes, active learning provides a principled way to select the next experiments that are maximally informative [82–84]. Surrogate models (e.g., Gaussian processes or neural predictors) can be updated with sparse measurements and used to propose new variants that balance exploration and exploitation. In practice, these approaches are frequently combined with library-based exploration (focused mutagenesis around hotspots) to accelerate convergence. Recent machine learning-guided directed evolution paradigms further illustrate how Bayesian optimization coupled with automated experiments can operate under real laboratory constraints, providing an algorithmic template that is closer to industrial optimization workflows [86–88].

### Automation and self-driving labs: Scaling DBTL in time and throughput

When the DBTL cadence shifts from monthly to daily or weekly iterations, automation becomes a prerequisite rather than a convenience. Self-driving laboratories integrate robotic liquid handling, standardized protocols, and data-management pipelines so that the transition from sequences to functional readouts becomes fast and reproducible. For binder programs, automation can be deployed at multiple stages: rapid DNA assembly and expression screening, standardized purification and SEC profiling, high-throughput display, fluorescence-activated cell sorting or microfluidic assays, and semiautomated SPR or BLI measurements for selected candidates [86]. Parallel efforts are pushing DBTL toward more robust and standardized implementations. One example is the coupling of cell-free expression and assembly with cell-free assays to reduce turnaround time and experimental variability, thereby enabling more stable learning signals for closed-loop optimization [87].

### A minimal experimental validation set: Anchoring closed-loop learning with high-quality readouts

A closed loop does not mean replacing everything with high-throughput assays. In contrast, the more high-throughput readouts one collects, the more a strict minimal validation set is needed to suppress false positives and ensure comparability across batches. For binders, this set should typically cover at least 3 types of information: (a) kinetics and affinity from physical gold-standard assays (SPR or BLI), reporting fitted *k*_on_, *k*_off_, and *K*_D_ rather than merely qualitative binding signals; (b) mechanism and epitope consistency, such as competition binding, epitope binning, or competition with a natural ligand or known antibody to confirm the intended epitope; and (c) developability baselines, such as SEC monodispersity, thermal stability, and aggregation propensity, to avoid mistaking nonspecific aggregators for strong binders [22]. In Table 3, we propose a minimal yet rigorous validation framework. To implement this practically, we recommend selecting a strategic subset of candidates (e.g., 10 to 50 top hits, supplemented with random edge cases for model calibration) for high-quality orthogonal validations, such as SPR, BLI, or ITC. By anchoring high-throughput ranking to this high-fidelity data, and applying complementary mitigations like batch correction and rigorous counter-screens, model updates in the closed loop can avoid being systematically driven off course by assay noise [89–91].

## Representative Workflows and Illustrative Case Studies

Building on the modules above, *de novo* binder design has converged on an increasingly standardized engineering workflow: geometric generation, sequence design, structural screening, and experimental closed-loop optimization [92,93]. Below, we highlight several representative scenarios that illustrate how this paradigm is implemented in practice.

### Epitope-focused binders: From epitope specification to specificity validation

The core advantage of epitope-focused design is that the spatial constraint of the target epitope is explicitly encoded in the generative process. In classic settings such as the SARS-CoV-2 receptor-binding domain, *de novo* miniprotein binders have demonstrated that epitope constraints and hotspot conditioning can yield compact, high-affinity binders that recapitulate desired contact patterns [94]. For example, initial computational screens for these targets yielded experimental hit rates of roughly 1% to 2% from tens of thousands of candidates, which were subsequently matured to subnanomolar or picomolar affinities (often reaching *K*_D_ values of 1 to 100 pM) via yeast display optimization [94]. Beyond affinity, epitope-focused workflows naturally support mechanistic validation: competition assays, epitope binning, and structure determination can verify whether binding occurs at the intended site [95]. Importantly, medicinally relevant performance often hinges on selectivity among homologs. Case studies on selective miniprotein inhibitors that discriminate among closely related integrins (e.g., α_v_β_6_ vs. α_v_β_8_) highlight that design goals extend beyond merely enhancing binding affinity to ensuring target selectivity and the desired mechanism of action [94–96]. Crucially, these case studies also reveal common experimental pitfalls. High enrichment scores in multivalent display formats frequently fail to translate to low *K*_D_ values in monomeric biophysical assays (e.g., SPR or BLI). This discrepancy often arises from avidity effects, confounding by expression levels, or polyreactive sequences prone to aggregation that appear enriched during selection. As a result, rigorous downstream validation, such as soluble expression screening, SEC, and monomeric binding assays, is required to confirm true binding affinity and specificity.

### Team- and industry-scale pipelines: Scalable generation, data feedback, and front-loaded engineering metrics

When binder design is deployed at team or industrial scale, the focus shifts from the feasibility of design to the reliability and scalability of candidate delivery. First, generation is scaled across multiple targets and tasks in parallel, often with standardized constraints and a shared screening funnel. Second, data feedback becomes a central asset: experimental outcomes and failure modes are systematically logged to recalibrate filters and improve subsequent rounds of design. Third, engineering metrics are considered earlier in the workflow, including expression, solubility, monodispersity, and manufacturability, so that late-stage failures can be reduced. Crucially, scale does not imply blindly increasing compute: reusable pipeline frameworks that modularize key steps (from random initialization to stable complexes) and enforce consistent evaluation can be more impactful than raw compute alone [92,97].

### Translational and clinically relevant examples: Stability advantages and real-world challenges

In translational settings, the most prominent advantage of *de novo* proteins is often not merely to surpass antibody performance but enabling new delivery and formulation windows through small size and high stability. Examples include cytokine mimetics and engineered binding proteins that modulate IL-2 and IL-15 pathways, where compact, stable scaffolds can support alternative dosing strategies and product formats [98]. At the same time, real-world challenges remain: immunogenicity risk, pharmacokinetics and clearance, manufacturing robustness, and the need for rigorous specificity and safety profiling [99]. Platforms such as EndoTags further illustrate that binding is only a starting point. Successful products must also couple binding to desired intracellular trafficking, uptake, or functional payload delivery, raising additional constraints that must be co-designed with the binding interface [100].

## Limitations and Future Directions

### Flexible interfaces and induced fit: Bridging the gap beyond static modeling

Current generation and complex-prediction pipelines are still largely built around single-structure snapshots. For targets that require substantial induced fit, loop rearrangement, or conformational selection (e.g., GPCRs, ion channels, or intrinsically flexible signaling proteins), static modeling can yield high-confidence yet mechanistically incorrect poses. Future progress will likely require explicit integration of conformational ensembles and dynamics into design: multistate generation and screening, ensemble-aware confidence metrics, and efficient approximations that incorporate flexibility without sacrificing throughput. In practice, a promising direction is to combine fast predictors with targeted MD or accelerated-sampling checks for a small subset, and to embed dynamic stability proxies into the screening funnel so that binder programs can achieve interpretable improvements on flexible and dynamic targets [101].

### Data scarcity and bias: Expanding the structural coverage of the PDB

Given the inherent resolvability bias of PDB data, the key is no longer to repeatedly restate the existence of bias, but to expand the boundaries of supervision signals. Strategies include incorporating cryo-electron tomography and other in situ structural modalities, leveraging crosslinking and mass-spectrometry constraints, and using high-throughput functional labels (DMS or display-enrichment data) to complement dynamic and context-dependent phenotypes. Equally important is to establish stricter out-of-distribution benchmarks and split protocols so that models are evaluated on their ability to generalize beyond the PDB’s coverage, rather than on memorizing interface patterns already present in structural databases.

### Multiobjective developability optimization: Pareto frontiers and context-aware decision making

Although the “Design objectives: Multidimensional trade-offs among affinity, specificity, and developability” section highlighted the necessity of multiobjective trade-offs, current engineering practice still often relies on simple linear weighting or threshold-based filtering [102]. A key future direction is to treat binder design explicitly as Pareto optimization: rather than collapsing objectives into a single scalar score early, pipelines can maintain a diverse set of candidates that spans the Pareto frontier across affinity, specificity, stability, solubility, expression, and immunogenicity [103]. Selection can then be adjusted according to the clinical scenario. For example, acute dosing may prioritize high affinity, whereas chronic dosing may place greater weight on low immunogenicity and long-term stability. This approach helps avoid discarding potentially valuable candidates too early [104,105].

### End-to-end complex generation and hybrid physics–generation systems

AF3 and RFAA bring protein–protein, protein–nucleic-acid, and protein–small-molecule interactions into a more unified all-atom framework, providing a foundation for end-to-end complex design and evaluation [17,18]. An emerging trend is to fuse generative sampling with explicit physical priors: use diffusion or other generative models to propose candidates under complex constraints, and then apply interpretable physics-based checks, including energy functions, stereochemical filters, and short-time dynamical stability proxies, to suppress high-confidence false positives. More broadly, hybrid systems that combine learned potentials with lightweight dynamics or energy refinement may improve transferability across chemical environments and assay conditions without sacrificing the throughput required for practical design funnels [101].

### Open data and methodological standardization: Building an ImageNet for protein design

Binder design still lacks a unified, ImageNet-like benchmark that enables direct, fair comparison across methods. Differences in targets, constraints, thresholds, and experimental conditions across laboratories can make reported successes appear incomparable and can hide false positives behind incompatible metrics. To enable cumulative progress, the field needs the following: standardized task definitions and split protocols; shared baseline pipelines; and open, reproducible implementations. To directly address these comparability issues, recent community competitions, such as Adaptyv Bio’s binder-design challenges, have begun to evaluate diverse methods against the same target under standardized experimental conditions [106]. These efforts, alongside computational benchmark suites (e.g., OpenStructure) and reproducible modeling implementations (e.g., OpenFold), provide key infrastructure for making binder-design research more comparable, reproducible, and scalable [107,108].

## Conclusion

Recent advances in structure prediction, inverse folding, and generative modeling have substantially expanded the scope of *de novo* binder design. It is now possible to generate candidate binders under structural constraints, evaluate them with increasingly informative computational filters, and refine them through iterative experimental feedback. At the same time, several limitations remain unresolved. Flexible targets, induced fit, data bias, uncertainty calibration, and developability trade-offs continue to limit the reliability of purely computational prioritization. For this reason, the most effective workflows are likely to be those that combine generative design with careful structural evaluation and experimentally grounded feedback. Overall, the field is moving toward more integrated design pipelines in which generation, screening, and validation are more closely connected. Continued progress in benchmarking, model calibration, and closed-loop optimization will be important for improving the reproducibility and practical utility of *de novo* binder design in both research and translational settings.
